# The Influence of the Chemical Composition of Flax and Hemp Fibers on the Value of Surface Free Energy

**DOI:** 10.3390/ma17051104

**Published:** 2024-02-28

**Authors:** Barbara Romanowska, Wanda Różańska, Małgorzata Zimniewska

**Affiliations:** Institute of Natural Fibers and Medicinal Plants National Research Institute, Wojska Polskiego 71B, 60-630 Poznan, Poland; wanda.rozanska@iwnirz.pl (W.R.); malgorzata.zimniewska@iwnirz.pl (M.Z.)

**Keywords:** flax fibers, hemp fibers, surface free energy, wettability, lignin, pectin, cellulose, hemicellulose, waxes and fats

## Abstract

The article presents the exploration of flax and hemp fibers’ surface free energy depending on the chemical composition of the fiber, which is closely related to the plant variety and the method of extracting the fiber. For this purpose, tests of the surface free energy (SFE), evaluation of the percentage content of individual fiber components and FTIR analyses were conducted. The research was carried out with the use of fibrous materials prepared in three different ways: 1. To analyze the effect of subsequent stages of flax fibers refining process on chemical composition and SFE, 2. to explore the dependence of fiber SFE on hemp variety, the water-retting hemp fibers were used, 3. To evaluate the influence of the retting method of hemp fibers BIAŁOBRZESKIE variety on SFE, the fibers extracted with the use of dew and water retting were used as the research material. The study confirmed that the content of individual components in the fiber influenced its sorption capacity and therefore determined its hydrophilic properties. The values of Pearson’s linear correlation coefficients determined in the statistical analysis proved that the surface free energy was strongly correlated with the content of individual components in the fibers. Understanding the wettability characteristics of bast fibers will allow modeling the properties of products made of these fibers and designing surface modification processes in order to obtain specific functionality of textile products, depending on their intended utilization.

## 1. Introduction

The study of surface free energy of all kinds of fibers takes on a new context. Depending on the application, the modification of surface tension can be used to improve moisture removal from the skin layers [1] and prevent the harmful effects of global warming by condensing water from the air in dry areas [2] or in the production of biocomposites [3]. For this reason, linen and hemp fibers are becoming more and more popular. These fibers are high-strength, biodegradable, and friendly to humans and the environment. Understanding the wettability mechanisms of flax and hemp fibers will enable better adaptation of this natural raw material to modeling and optimization of production processes and obtaining high-quality products.

Flax and hemp plants provide natural lignocellulosic fibers. Due to their specific properties, these bast fibers are used for the production of clothing, decorative, interior and upholstery products, as well as insulating materials and as reinforcement of composites and biocomposites [4]. Clothing made of flax and/or hemp fibers creates a specific synergy with the user’s skin, guaranteeing physiological comfort and protecting the body against the harmful effects of UV rays [5]. Clothing made of flax/hemp fibers is characterized by good air permeability, allowing the skin to breathe freely. In addition, they support the natural immunity of the skin, by ensuring a favorable microclimate, they reduce the growth of reactive oxygen forms and antioxidant stress in the human body [6] and do not cause excessive fatigue of the wearer. Due to the content of phenolic acids in their chemical composition, which are known as strong antioxidants, clothing made of flax/hemp fibers contributes to the neutralization of free radicals appearing next to human skin, protecting against their adverse effects. The fibers are also characterized by antibacterial properties. However, on the other side, they have the ability to keep the bacterial microflora of the skin on the proper level [7,8,9]. Depending on the variety of flax and hemp plants, as well as on the method of extracting the fiber from straw, the fibers show different chemical compositions. Flax and hemp fibers contain cellulose, lignin, pectin, hemicellulose, waxes and fats. Each of the individual fiber components has different wetting properties; hence, the wettability of the fibers is diversified [10,11,12].

**Figure 1:**
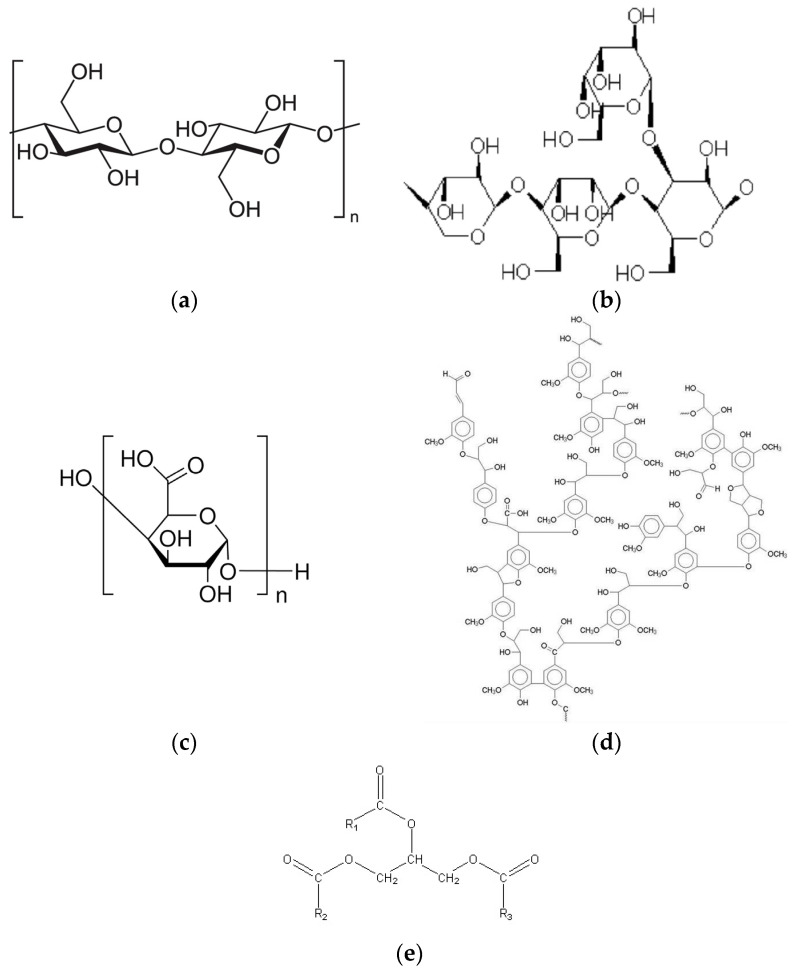
Structure of: (**a**) cellulose [20], (**b**) hemicellulose [21], (**c**) pectin [22], (**d**) lignin [23], (**e**) waxes and fats [24].

The fiber components that have hydrophilic properties are celluloses, hemicelluloses and pectin, while the hydrophobic components are lignin, waxes and fats [13]. Cellulose is a polymer composed of linearly connected segments with a significant number of free hydroxyl groups (Figure 1a) which contribute to the hydrophilic nature of cellulose [14,15]. Hemicellulose contains the hydroxyl groups in the hemicellulose chain (Figure 1b) providing hygroscopic properties to the hemicellulose [16]. Pectin (Figure 1c), like hemicellulose, is a polysaccharide that causes an increase in the water sorption capacity of the fiber, enhancing the hydrophilic nature of fiber [12,14]. Lignins (Figure 1d) are hydrophobic, chemically inactive and resistant to enzymatic activity [17]. On the other hand, waxes and fats (Figure 1e) are insoluble in water, and have hydrophobic properties [18,19].

The chemical composition of flax/hemp fibers determines their hydrophobic/hydrophilic properties including facility for wettability. The surface free energy of natural fibers is the subject of many studies [9,25,26,27,28,29,30,31,32], most often in the case of their application to reinforce composite materials, where the wetting ability is an important feature from the point of view of the composite forming process, because the surface free energy determines the adhesion between the fiber and the polymer matrix.

The specificity of the fiber morphology, characterized by surface heterogeneity, creates some difficulties with the repeatability of the measurement and in consequence with the clear analysis of the surface free energy test results. Therefore, different measurement methods are used which, despite the differences in the technique of execution, ensuring comparable results. Heng et al. claim that the best method is Inverse Gas Chromatography (IGC), because this method is not dependent on the inconvenience of using methods based on measuring liquids [25]. However, usually, the Wilhemy method is used, in which the contact angle is measured by immersing the sample in the measuring liquid. This method was used, for example, by Velde and Kiekens, who analyzed the hysteresis curves of the contact angles in various measuring liquids applied to flax and glass fibers used as reinforcement of thermoplastics [26]. The capillary growth method, which is based on the dynamics of liquid adsorption measured by the capillary growth height, was used by Gassan et al. Researchers using this method in their work proved that higher polarization of natural fibers can be achieved through the use of the silanization process [10]. An alternative technique to the Wilhelmy method was proposed by Schellbach et al., who used the technique of testing the contact angle resulting from the meniscus created by liquid locked in between two parallel natural fibers kept at a specific distance on a Teflon frame [33]. Xu et al. applied the Drop on Fiber method to evaluate the wettability of bamboo fibers treated with argon plasma to improve the natural dyeing process [34].

The study of the surface free energy of natural fibers has become noteworthy for the design and optimization of technological processes of fiber modification depending on the purpose of the fibers, e.g., for technical applications such as composite materials as well as for apparel purposes including functional clothing, where ensuring adequate physiological comfort by improving moisture transport plays a key role. However, the key is a thorough understanding of the wettability mechanisms and the characteristics of flax and hemp fibers in terms of the free surface energy.

The aim of this study is to investigate the effect of the chemical composition of flax and hemp fibers from various varieties of fibrous plants on their surface free energy.

To realize the purpose of the research, three varieties of flax fibers were subjected to the refining process to assess the process effect on SFE of the fibers, as well as four varieties of hemp fibers extracted using the water-retting method to evaluate fibrous plan variety effect on fibers SFE, and BIAŁOBRZESKIE variety hemp fibers obtained using the dew- and water-retting methods to investigate the retting method effect on fibers free surface energy. Each type of obtained fiber was tested for surface free energy using the Sessil Drop method. Additionally, the percentage content of individual components of the fibers was determined and the characteristics of the fiber structure were analyzed using FTIR.

The research is a continuation of previous work on the Antioxidant Potential of Hemp and Flax Fibers Depending on Their Chemical Composition [7].

## 2. Materials and Methods

### 2.1. Materials

The flax straw of the Polish varieties MODRAN, NIKE and B14 and the hemp straw of the Polish varieties BENIKO, WOJKO, TYGRA and BIAŁOBRZESKIE were selected for the tests.

The plants were cultivated at the same growing season, flax and hemp straw came from the Polish plantations conducted at the experimental stations of the agriculture research institutes.

#### 2.1.1. Flax Fibers Preparation

In order to determine the fiber chemical composition of each flax variety MODRAN, NIKE and B14 and to study the fiber surface free energy in the context of their chemical structure, the fibers were extracted from the collected straw with the use of a decortication process. Decortication is a mechanical process of breaking and removing the woody part of the stem targeting extraction of one-type fibers. After decortication, the fibers were degummed with the use of a hydrodynamic process, dried and then cottonized.

At the initial stage of processing flax straw, the decortication process was used, which consisted of the mechanical separation of the straw from the fibers with the use of a specially adapted process line for decortication [35]. The decortication delivers bundles of fibers glued together with fixed woody parts of the stem, resulting in a large amount of impurities, the decorticated fibers are not suitable for application in the spinning process and the production of textiles. Therefore, it is necessary to apply subsequent refining processes to improve the quality of the fibers. The decorticated fibers were subjected to the process of hydrodynamic degumming (process duration: 24 h, water temperature: 30 °C), supported by ultrasounds. The fibers after degumming and drying were subjected to the cottonization process, which resulted in reaching the flax fiber parameters of length and linear density similar to that of cotton [36]. Decorticated degummed cottonized fibers were used to investigate the relationship between flax variety and its chemical composition and free surface energy.

#### 2.1.2. Hemp Fibers Preparation

A comparative analysis of the chemical composition of the hemp fiber and the surface free energy of these fibers, depending on the variety of hemp, was carried out for the BENIKO, WOJKO, TYGRA and BIAŁOBRZESKIE varieties. The fibers were extracted from the straw with the use of water retting, while conditions of the process were the same for each hemp variety. The water retting of hemp samples was conducted under laboratory conditions (process duration: 120 h, water temperature: 34 °C).

The influence of the extraction method on the chemical composition of the fibers and their surface free energy was investigated with the use of the BIAŁOBRZESKIE variety for which two retting methods were used:

- dew retting in the field for a period of 7 weeks until they were fully retted,
- water retting under laboratory conditions (process duration: 120 h, water temperature: 34 °C).

The dew and water-retted straw of each sample of tested hemp after drying was processed on a laboratory turbine in order to reduce the woody parts of the stem and obtain pure fibers.

### 2.2. Method

#### 2.2.1. Chemical Analyses

Tests on the percentage of individual components of flax and hemp fibers, i.e., waxes and fats, pectin, lignin, cellulose and hemicellulose, were carried out on the basis of normative documents and gravimetric methods developed and validated by the Institute of Natural Fibres and Medicinal Plants National Research Institute:

- Waxes and fats content (%), was measured according to the Polish Standard no. BN-86/7501-10 [37]. The percentage content of wax and fat substances was determined by extracting them with an organic solvent (petroleum ether) in a Soxhlet extractor and weighing the residues after vaporization of the solvent.
- Lignin content (%) was determined according to the Polish Standard BN-86/7501-11 [38]. The lignin content was measured by dissolving cellulose, hemicellulose and pectin with a mixture of concentrated sulphuric and ortho phosphoric acids, followed by draining off the remaining insoluble lignin.
- Pectin content (%) tests were conducted by a gravimetric method according to a method developed at the Institute of Natural Fibres and Medicinal Plants National Research Institute. The percent share of pectin was determined by dissolving them in ammonium citrate and then precipitating from the solution with calcium chloride and by measuring the weight of the calcium pectinate precipitated from the solution.
- Hemicellulose content (%) in the flax end hemp fiber was determined according to the Polish Standard BN-77/7529-02 [39]. The hemicellulose content was measured by dissolving the hemicellulose present in the fiber with a 1% solution of sodium hydroxide, filtering off the residue after dissolution, drying it and weighing it. Then the hemicelluloses were calculated from the mass loss of the sample.
- Cellulose content (%) in flax and hemp fiber was measured according to the Polish Standard no. PN-92/50092 [40]. The cellulose content was measured by dissolving lignin and other substances present in the fiber with a mixture of acetylacetone and dioxane, acidified with hydrochloric acid.
- Fourier transform infrared spectroscopy (FTIR) with an ATR (Attenuated Total Reflectance) attachment was performed in TA Instruments the iS10 model with Smart iTX ZnSe cristal. The spectrum of the fibers contained 16 scans per second at a resolution of 4 cm^−1^ within the range from 600 to 4000 cm^−1^.

#### 2.2.2. Surface Free Energy Test

The surface free energy tests were carried out by measuring the contact angle on the Goniometer DSA30S by Krüss GmbH (Hamburg, Germany). The apparatus uses the Sessile Drop method, which involves “placing” a drop on the surface of the sample [41]. The resulting drop shape image is recorded and analyzed by specialized software (Advance, v. 1.9.0.8, Krüss GmbH, Hamburg, Germany). Then the value of the surface free energy is calculated from the Owens–Wendt equation:

$$\gamma_{s}=\gamma_{s}^{d}+\gamma_{s}^{p}$$

where:

$\gamma_{s}$—surface free energy,
$\gamma_{s}^{d}$—geometric mean of dispersive components,
$\gamma_{s}^{p}$—geometric mean of polar components [42].

Two measuring liquids are used to measure the contact angle: polar (HPLC grade water) to obtain polar components, and a non-polar (diiodomethane), thanks to which dispersive components are obtained. The surface free energy of a solid (in this case the fibers) is calculated as the sum of the means geometric dispersive and polar components.

**Figure 2:**
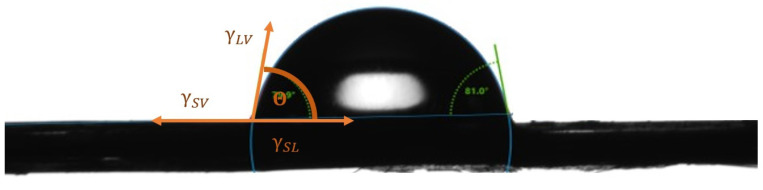
A drop formed by the Sessile drop technique on the surface of a flax fiber. Photo: Romanowska, B.

Mean geometric components arise as a result of the contact of a solid body (i.e., fibers) with the dispersive and polar measuring liquids. The contact of the solid body with the measuring liquid causes the meeting of three phases, thanks to which geometric components are obtained: γ_SL_—on the border between fiber and measuring liquid, γ_SV_—on the border between fiber and air, γ_LV_—on the border between the measuring liquid and air, this is visible on Figure 2.

The surface free energy of the geometric component is determined on the basis of the surface free energies of the solid body contact with the measuring liquid according to Young’s equation:

$$\gamma_{S}=\gamma_{SL}+\gamma_{L}cos\Theta$$

where:

$\gamma_{s}$—surface free energy of solid,
$\gamma_{SL}$—surface free energy of solid in contact with the measuring liquid,
$\gamma_{L}\;$—surface free energy of the measuring liquid [43].

#### 2.2.3. Statistical Analyses

The statistical analysis was performed using the STATISTICA software (8, TIBCO Software Inc., Palo Alto, CA, USA). Data were expressed as mean value and standard deviation (SD) for each measurement. The statistically significant differences between the fibers were assessed by the one-way analysis of variance (ANOVA) and Tukey’s Honest Significant Difference (HSD) test for *p* < 0.05. The correlation analysis was performed due to the ability to identify the linear relationship between two variables: the content of individual chemical components of the fiber and the value of the surface free energy. Pearson’s linear correlation coefficients were determined in order to evaluate the strength of interdependencies between the variables. In order to provide a more accurate substantive interpretation, the obtained results of the correlation coefficients in the form of linear functional relations with the indication of confidence intervals at the level of 95% were presented.

## 3. Results and Discussion

### 3.1. Chemical Analyses

The main component of flax and hemp fibers of the whole chemical fiber composition is cellulose [7,44], which accounts for about 64–78%. The fibers also contain hemicellulose (approx. 13–22%), lignin (approx. 2–9%), pectin (approx. 0.5–5.5%), waxes and fats (approx. 0.2–1.8%).

The results of previous research confirmed that the chemical composition of flax and hemp fibers is different for the tested plant varieties [7]. Additionally, in the case of flax, the chemical composition of the fiber changes as a result of the application of subsequent fiber treatment processes. In the case of hemp, the chemical composition of the fiber varies depending on the retting method used, Table 1, Table 2 and Table 3.

#### 3.1.1. FLAX Fiber Chemical Composition in Relationship of Plant Variety and Subsequent Stages of the Applied Technological Chain

The analysis of individual compounds contained in the flax fiber was conducted based on test results shown in Table 1.

Cellulose is responsible for the mechanical, physicomechanical and sorptive properties of the fiber [13,14]. Refining processes lead to the removal of pectin, lignin and hemicellulose from the fibers resulting in the exposure of cellulose, which is dominantly responsible for the hydrophilic properties of bast fibers [45].

It was found that the cellulose content in the flax fiber increased after the application of the wet degumming process regardless of the tested variety and is significantly higher compared to the decorticated fiber. The third process, i.e., cottonization, caused a reduction in the cellulose content in the flax fiber of the NIKE and B14 varieties. In the case of the MODRAN variety, the cellulose content did not differ statistically.

Hemicellulose is an amorphous heteropolymer that enhances the vertical orientation of the fibers in the plant [16]. It has a hydrophilic nature [46]; however, technological processes lead to its partial degradation [47]. The results of the test of the hemicellulose content in the flax fiber showed that the use of hydrodynamic degumming for the decorticated fiber and further cottonization significantly influenced the removal of the hemicellulose component from the fiber. The above relationship was demonstrated for all three tested varieties of flax. The comparison between the flax cultivars showed that NIKE was characterized by the significantly lowest content of hemicellulose in the fiber after the process of hydrodynamic degumming and cottonization of decorticated flax, i.e., 13.84% compared to MODRAN and B14. Hemicelluloses have a lower degree of polymerization (SP < 300) than cellulose, lower regularity of structure and degree of structure order, which makes them less resistant to degradation [48]. Therefore, in the subsequent technological processing stage, the content of hemicellulose in the fiber decreased.

Pectin acts as a link between cells in the fiber resulting in the joining of the woody part and fibers together. Pectin, similar to hemicellulose, is a polysaccharide and acts as fiber reinforcement, ensuring the stability of plant tissues [12,14]. The pectin is removed during fiber extraction to separate fibers from the stem and to divide the technical fibers [49,50]. The pectin content in the decorticated flax fiber in all tested varieties was determined at a similar level, above 4%. However, the further processing of the decorticated fiber using the wet degumming and cottonization methods caused a reduction in fiber with a lower pectin content in the fiber, which is well visible for the NIKE. Pectin content in B14 is the highest for wet degummed fibers although after cottonization the component content reached the lowest value. It can be explained that the pectin that should be leached during the wet process is still on the fiber surface and cottonization causes chipping of the loose pectin from the fiber surface. In the case of the MODRAN variety, no significant influence of further refinement of the decorticated fiber on the pectin content in the fiber was demonstrated. The biggest amount of pectin in the fibers after the application of the whole technological chain was found in MODRAN variety fibers at 4.72%. In the case of B14 pectin content, it was 3.57% and the lowest value was shown for NIKE—2.39%.

Waxes and fats protect the plant against microbes and water loss. In spinning processes, they reduce the coefficient of friction, facilitating the formation and thinning of a stream of parallelly arranged fibers [18,19]. Various methods of fiber processing lead to partial removal of waxes and fats, but may lead to better fiber adhesion [51,52,53]. The evaluation of the content of waxes and fats in the flax fiber of the MODRAN and NIKE variety showed that the degumming of the decorticated fiber caused the partial removal of waxes and fats in the fiber as a result of the combined action of water and ultrasound. Waxes and fat contents in flax fibers variety B14 increased in the fiber after subsequent technological stages and were highest comparing NIKE and MODRAN after degumming and cottonization. B14 flax fiber is derived from a variety bred on the basis of genetic engineering, hence its behavior in the technological chain differs from traditional flax and is difficult to explain at this stage of the work.

Lignin protects the plant against mechanical damage and pathogens. They increase the stiffness of the fibers and reduce their flexibility [17]. The high content of lignin hinders the processing of fibers and creates difficulties with their spinnability. However, their presence is desirable when it is important to increase hydrophobicity by reacting with synthesized modifying compounds [54,55,56]. The analysis of the results of testing the content of lignin in the decorticated flax fiber showed that the lowest lignin amount is characteristic of the decorticated flax fiber of MODRAN, i.e., 4% compared to the other tested varieties. The lignin content was 8.6% for the NIKE variety and 5.25% for the B14 variety. Further processing of the MODRAN variety fiber with the use of hydrodynamic degumming or cottonization did not significantly affect the lignin content in the fiber. On the other hand, it was observed that the application of hydrodynamic degumming of the decorticated fiber of the B14 variety increased the lignin content, i.e., 6.69%, and in the case of the NIKE variety, the lignin content was the lowest and amounted to 4.49%. Applying the cottonization process did not significantly change the lignin content in the fiber for all the varieties tested.

It can be assumed that the application of the wet degumming process for the decorticated fiber significantly changes the chemical composition of the tested fiber and varies for different varieties of flax.

As revealed in the above research, in the case of tested flax varieties, the refinement of the decorticated fiber by the method of hydrodynamic degumming and then cottonization allowed us to obtain a fiber with a higher cellulose content and a lower hemicellulose content, compared to the fiber processed only by decortication, regardless of the variety used. For the other fiber components, the refinement of decorticated flax fiber of the NIKE and MODRAN varieties, reduced the content of waxes, fats and pectin in the fiber. The NIKE variety fiber also has a reduced lignin content as a result of the hydrodynamic degumming process. However, considering the influence of the hydrodynamic degumming process on the content of waxes and fats, pectin and lignin in the case of the B14 variety and lignin in the case of the MODRAN variety, these relationships were not so unequivocal.

#### 3.1.2. HEMP Fiber Chemical Composition in Relationship of Plant Variety

The advantage of water retting is that it takes place in completely controlled conditions, which makes it superior to the dew-retting method [57]. It also allows obtaining higher quality fiber by removing non-cellulosic components for spinning applications. However, the disadvantage of water retting is the negative impact of the process on the environment due to the consumption of large amounts of water and excessive production of water pollutants [58]. Nevertheless, thanks to the controlled parameters of the process, reliable results can be obtained which allows the evaluation of differences in fiber chemical composition depending on the fiber varieties.

The analysis of the chemical composition of fibers obtained from four varieties of hemp with the use of water retting was conducted based on results presented in Table 2. The result of tests confirmed that the highest cellulose content 72.53% was found in the WOJKO variety compared to the other varieties. The obtained differences between the cellulose content in other varieties of hemp subjected to water retting are at the level of 0.5–1.74% and are statistically insignificant.

The comparison of the tested hemp varieties showed that the fiber of the BIAŁOBRZESKIE variety contained the lowest hemicellulose content. The difference between the individual cultivars ranged from 0.63% to 2.3% and was statistically significant confirmed by the Tukey test.

The content of lignin in the fiber of all tested varieties ranged from 2.38% to 3.02% and did not differ significantly, which was statistically shown by the Tukey test.

The lowest pectin content, i.e., 0.56%, was characteristic of hemp fiber of the TYGRA variety and the highest of the BENIKO variety, i.e., 1.47%. The obtained results were statistically significant confirmed by the Tukey test.

For hemp fibers of the BENIKO, WOJKO and TYGRA varieties were characterized by a lower waxes and fats content, i.e., at a similar level of 0.24–0.25% compared to the BIAŁOBRZESKIE variety.

#### 3.1.3. HEMP Fiber Chemical Composition in Relationship of Applied Retting Method

Comparison of dew and water-retting methods and the quality of fibers obtained using these methods has been the subject of literature [57,58,59,60,61,62]. Dew retting is widely used due to its low impact on the environment and economic conditions, but the course of the retting process depends on weather conditions [62]. The retting process on the field is difficult to control and may result in a diversity of fiber quality. Water retting is a short-term process under controlled conditions [57], but due to heavily polluted sewage, retting cannot be used in countries in Europe and other countries [61].

The analysis of the chemical composition of the BIAŁOBRZESKIE variety extracted with the use of water or dew retting was conducted based on test results shown in Table 3.

Results of the test of cellulose content in hemp fiber, the BIAŁOBRZESKIE variety, depending on the applied method of retting, showed that the water-retted fiber had a statistically significant higher cellulose content compared to the dew-retted ones, which was confirmed by statistical calculations performed with the Tukey test. This is due to the fact that water-soluble components such as pectin were removed, lignins were partially washed away and hemicellulose was partially degraded. Due to the removal of some of the components, the percentage of cellulose in the fiber increased.

Hemp fiber of BIAŁOBRZESKIE variety obtained using the water-retting method is characterized by a significantly lower content of hemicellulose in the fiber compared to the fiber obtained by the dew retting in the field. A comparison of the chemical composition of hemp fibers extracted with the use of the applied retting methods showed that the fiber obtained by the water retting is characterized by a significantly lower lignin content compared to the fiber obtained by the dew retting. It was also shown that pectin content in water-retted fibers was lower in comparison to dew-retted hemp fibers showing that water retting removes pectin from the fiber more effectively than dew retting due to the fact that pectin is partially soluble in water [57]. The tests showed that the BIAŁOBRZESKIE fiber obtained in the water-retting process is characterized by lower waxes and fat content compared to the fiber obtained by the field dew-retting method. In the case of hemp, the straw retting in water resulted in obtaining a fiber with a higher content of cellulose and a lower content of other components, compared to the fiber obtained with the use of the dew retting. The largest significant differences in the content of chemical components in the fiber were observed for pectin. The differences between the other components of the hemp fibers are rather small. The above phenomenon can be explained by the fact that the application of the water-retting method removes the components accompanying cellulose more effectively than in the case of retting in the field, and thus increases the cellulose content in the fiber.

The revealed dependences of the chemical composition of the fiber on the plant variety or the methods of retting hemp straw are also confirmed by the research of other authors [63,64]. The authors in their research indicated the link between the method of fibers obtaining and their further application. In the case of the textile industry, it is important to obtain a fiber of the required linear density, length, strength and purity, homogeneity guaranteeing optimal efficiency. This approach was associated with the removal of components accompanying cellulose, i.e., pectin, hemicellulose, lignin as well as waxes and fats. The authors also showed that the quality of the fiber depends on the variety used, which affects the structure of the monofilament itself in the plant, the microscopic features of the fiber, the physico-mechanical properties and the physico-chemical properties of the fiber.

In this work, in addition to the analysis of the chemical composition of the fiber determined with the use of chemical methods, the identification of compounds contained in the top layer of the fiber structure was carried out using the spectrophotometric analysis using the ATR-FTIR total internal reflection method.

**Figure 3:**
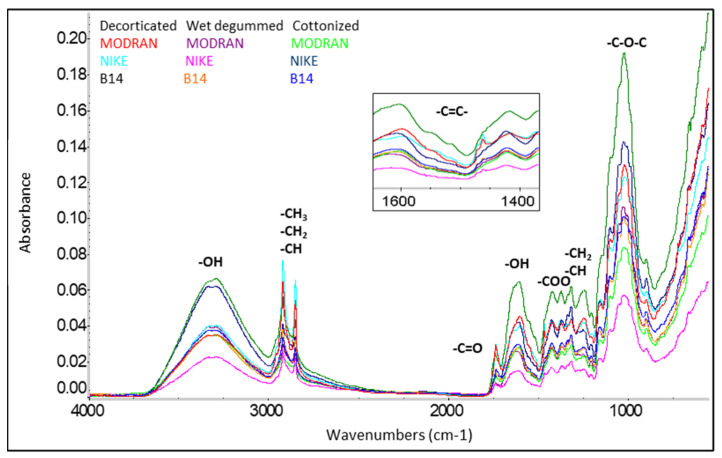
ATR/FTIR spectra of flax fibers [7].

**Figure 4:**
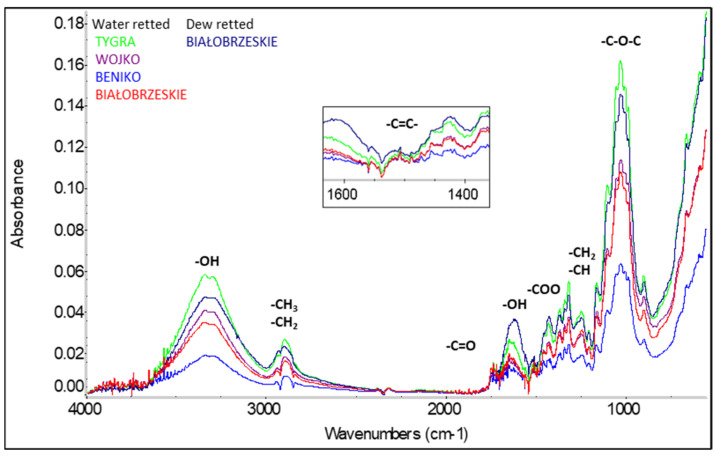
ATR/FTIR spectra of hemp fibers [7].

Based on the obtained infrared spectra (Figure 3 and Figure 4) in all tested flax and hemp fibers, the presence of bands in the wavelength range was identified, which correspond to the vibrations of polar bonds of functional groups such as: hydroxyl OH, carbonyl C=O, carboxyl COOH and ether C-O-C and vibrations of nonpolar functional groups such as: aromatic C=C and alkyl CH, CH_2_ and CH_3_ [7].

Analysis of the fiber structure showed that all identified bands are derived from the chemical components of the fiber, i.e., cellulose, hemicellulose, lignin, and pectin as well as waxes and fats. The detailed lists of characteristics of the main absorbance spectra in FTIR of tested fibers are shown in Table 4.

Cellulose is the largest structural component of bast fibers. This natural biopolymer consists of a large number of segments, D-glucose molecules linked linearly by β-1,4-glycosidic bonds. Aggregation in the cellulose macromolecule chain occurs through stable intramolecular chemically covalent bonds and weaker intermolecular bonds such as hydrogen bridges or van der Waals forces. Moreover, the presence of polar hydroxyl groups (-C-OH) and non-polar hydrogen groups (-C-H) makes the cellulose molecule amphiphilic. The hydroxyl groups are in the equatorial direction, so cellulose is hydrophilic in this plane. On the other hand, in the axial direction, there are C-H bonds which give the cellulose a hydrophobic character. The interactions of intermolecular bonds result in the formation of such an arrangement of cellulose chains, due to which cellulose forms a flat polymer chain with two surfaces with different polarizations. However, due to the strong affinity for the hydroxyl groups in cellulose, hydrophilic properties play a dominant role. The van der Waals intermolecular bonds occurring in the cellulose chain contribute to the increase in the adsorption energy at the equatorial hydroxyl groups directed outwards from the cellulose chains. The interaction of cellulose hydroxyl groups with water molecules is based on the principle of exothermic reactions, occurring spontaneously and caused by the randomness of the system, which is caused by the structural anisotropy of cellulose [15,65].

Hemicellulose is located in fibrous plants between lignin and cellulose. Chemically, hemicellulose is a polysaccharide composed mainly of pentosans and hexosans. Like cellulose, it contains hydroxyl groups along the entire length of the main and side chains of this polymer, which makes it hydrophilic. However, hemicellulose is a water-insoluble compound. In flax and hemp fibers, it forms strong complexes with cellulose and lignin. According to the literature [16,66], the nature of these bonds is so strong that they form chemical bonds with cellulose that are difficult to break. Hemicellulose contributes to the formation of bonds in the fiber, and in the mature phase the fiber is softened, which allows easier separation of fiber complexes [67,68].

On the other hand, pectin, similar to hemicellulose, belongs to polysaccharides; however, they differ significantly in their structure from hemicelluloses, because they are made of an acidic main chain (polygalacturonic acid), and there are neutral sugars in the side chain. Pectin, like hemicellulose or cellulose, contains hydroxyl groups that give the molecule hydrophilic properties. Also, protein residues and residues of some esters (e.g., methyl, acetyl) give pectin hydrophilic properties. Pectin in the plant is a factor that binds monofilaments, therefore during retting, it is most easily degraded [12,14,69,70]. This is due to the presence of hydrophilic carboxyl groups -COOH, due to which the molecule is characterized by a high ability to absorb water and swell.

Waxes and fats protect the fiber plant against water loss and prevent the entry of pathogenic microorganisms. In terms of chemistry, they are a diverse group of hydrocarbons, which include fatty acids containing hydrophilic ends. Despite the availability of hydrophilic groups (-CH, -CH_2_), waxes and fats are insoluble in water, which makes them hydrophobic. However, as a result of the initial method of soaking the straw in water, it is possible to remove them almost completely in mechanical processing [12,18,19,66].

Lignin keeps plant tissues of natural fibers reinforcing cellulose and hemicellulose. Lignin is made of a polymer that is part of the fiber cell wall. Lignin does not have a primary defined structure. The structure of lignin includes polycyclic aromatic hydrocarbons (responsible for the hydrophobic nature of lignin) also containing hydrophilic side groups, i.e., hydroxyl (OH) and methoxy (OCH_3_). Nevertheless, lignin is a component of the fiber with hydrophobic, water-insoluble properties [13,16].

The conducted research shows that spectrophotometric separation of individual compounds from the spectrum is impossible, due to the presence of the same functional groups in the case of cellulose, hemicellulose, lignin, and pectin.

### 3.2. Surface Free Energy Test

**Figure 5:**
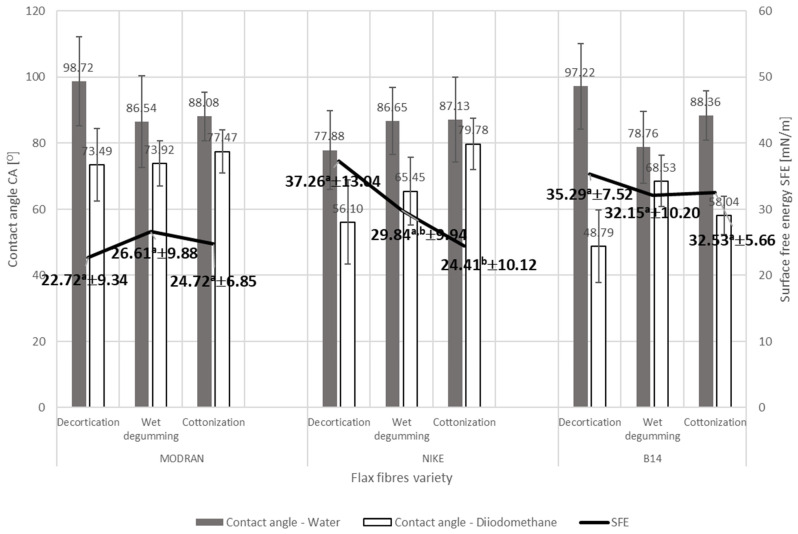
The results of the surface free energy determined by measuring the contact angle method of flax fiber varieties depending on the consecutive stage of fiber processing.

**Figure 6:**
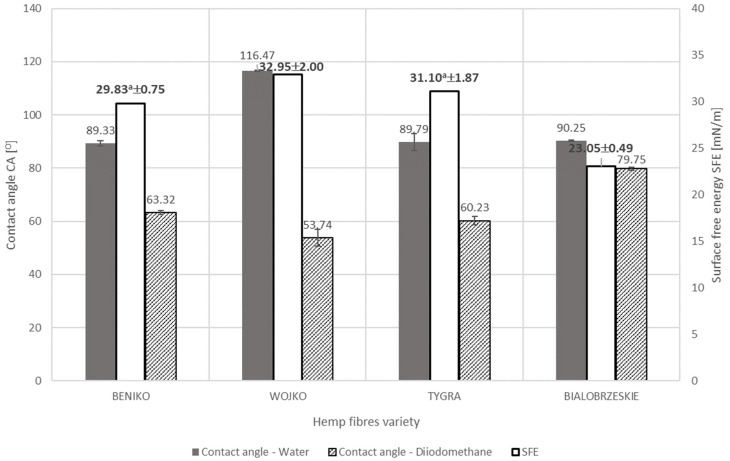
The results of the surface free energy determined by measuring the contact angle method of hemp fiber varieties BENIKO, WOJKO, and TYGRA obtained by the water-retting method.

**Figure 7:**
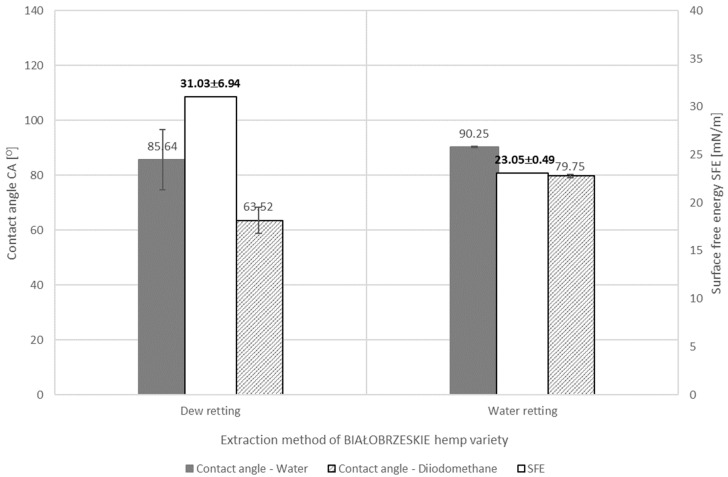
The results of the test of BIAŁOBRZESKIE hemp fibers’ surface free energy determined by measuring the contact angle method depending on the method of fiber extraction.

The diversity in the content of the fiber’s individual components, which have a hydrophilic or hydrophobic nature, causes the differing in the wettability of the fibers. Figure 5, Figure 6 and Figure 7 below show the results of the surface free energy measurement of the flax and hemp fibers coming from the tested plant varieties obtained with the use of various extraction methods and after each stage of decorticated fiber processing. The surface free energy was determined by the contact angle test method with two measuring liquids: polar (HPLC grade water) and non-polar (diiodomethane).

The obtained results of testing the surface free energy of flax and hemp fibers are consistent with the previously performed studies presented in the literature [9,25,26,27,28,29,30,31,32]. However, there are no reports in the literature on testing the surface free energy of bast fibers by the Sessile Drop method. Nevertheless, the authors of previous studies described in the available literature did not present an analysis of the surface free energy value of bast fibers depending on the chemical structure of the fiber, plant varieties, and the fiber extraction and processing process used.

The results of the test of the flax fibers’ surface free energy determined by measuring the contact angle method depending on the consecutive stage of fiber processing are visible in Figure 5. Results are expressed as mean value and uncertainty as a numerical value for the surface free energy value and as whiskers for the contact angles. Values sharing the same letter are not significantly different from each other (Tukey’s HSD, *p* < 0.05). Tukey’s test showed no significant differences between the results of surface free energy of flax fiber varieties depending on the consecutive stage of decorticated fibers processing. Nevertheless, the phenomenon of changes of differences between contact angle values obtained with polar and non-polar liquid was observed for flax fibers after each stage of the technological process. In the case of MODRAN and NIKE varieties of flax, the subsequent technological stage of flax fibers processing contributes to reducing the differences between the polar and the dispersive nature of the fibers, which is expressed in the difference in wettability angles of fibers tested with the use of the polar HPLC grade water and the dispersive diiodomethane. In the case of B14 fibers, this regularity is not such clear, because the contact angle created by diiodomethane is lower for cottonized fiber than degummed fiber resulting in bigger differences in contact angles between polar and dispersive liquids. However, the value of surface free energy of NIKE and B14 fibers decreased after subsequent stages of the technological chain. In the case of MODRAN variety, we cannot observe such regularity. The regularity of changes in contact angle and surface free energy is clearly visible for the NIKE variety, where increasing water contact angle and decreasing differences between polar and the dispersive nature of the fibers resulted in a decrease of surface free energy.

In the case of fibers derived from different varieties of hemp obtained by the use of the water-retting process, the influence of chemical composition on the wettability of the fibers was very clear. The results of the surface free energy determined by measuring the contact angle method of hemp fibers varieties BENIKO, WOJKO, and TYGRA obtained by the water-retting method are shown in Figure 6. Results are expressed as mean value and uncertainty as a numerical value for the surface free energy value and as whiskers for the contact angles. Hemp fiber of the BIAŁOBRZESKIE variety (Table 2) has the lowest total content of hydrophobic components, e.g., lignin, waxes and fats. The lowest content of hydrophobic components of the BIAŁOBRZESKIE variety is reflected in the smallest difference between the polar part HPLC grade water contact angle—90.25° and the dispersive part diiodomethane contact angle—79.75°, which is visible on Figure 6. The difference is 10.05° and it is the smallest of all the tested fibers, which results in the lowest surface free energy—23.05 mN/m. The highest surface free energy is characteristic of the fiber of the WOJKO variety—32.95 mN/m. WOJKO has the largest difference (62.73°) between the polar and dispersive part (HPLC grade water contact angle 116.47°, diiodomethane contact angle 53.74°), which is due to the highest total content of hydrophobic components among all considered hemp fibers. Statistically insignificant differences between values of surface free energy were observed only between hemp fibers of the BENIKO and TYGRA varieties.

The results of the research on the surface free energy of hemp fiber of the BIAŁOBRZESKIE variety extracted from straw with use of dew and water-retting methods confirmed that the extracting method affects the fiber chemical composition (Table 3), resulting in diversity of the sorption properties and the values of the surface energy of the fiber which was confirmed by the statistical analysis of significant differences Tukey’s test as well. The results of the surface free energy determined by measuring the contact angle method of BIAŁOBRZESKIE hemp fibers variety depending on the method of extraction are presented in Figure 7. Results are expressed as mean value and uncertainty as a numerical value for the surface free energy value and as whiskers for the contact angles. Statistical analysis Tukey’s HSD with *p* < 0.05 showed significant differences between the average values of the surface free energy of the tested fibers. The water-retting causes the binding of water molecules with the hydroxyl groups of the cellulose of the fiber which makes the polarization changes. In consequence, the water-retted BIAŁOBRZESKIE fibers showed higher contact angle for both liquids water and diiodomethane but lower differences between them and lower surface free energy in comparison to dew-retted fibers.

### 3.3. Statistical Analyses

**Figure 8:**
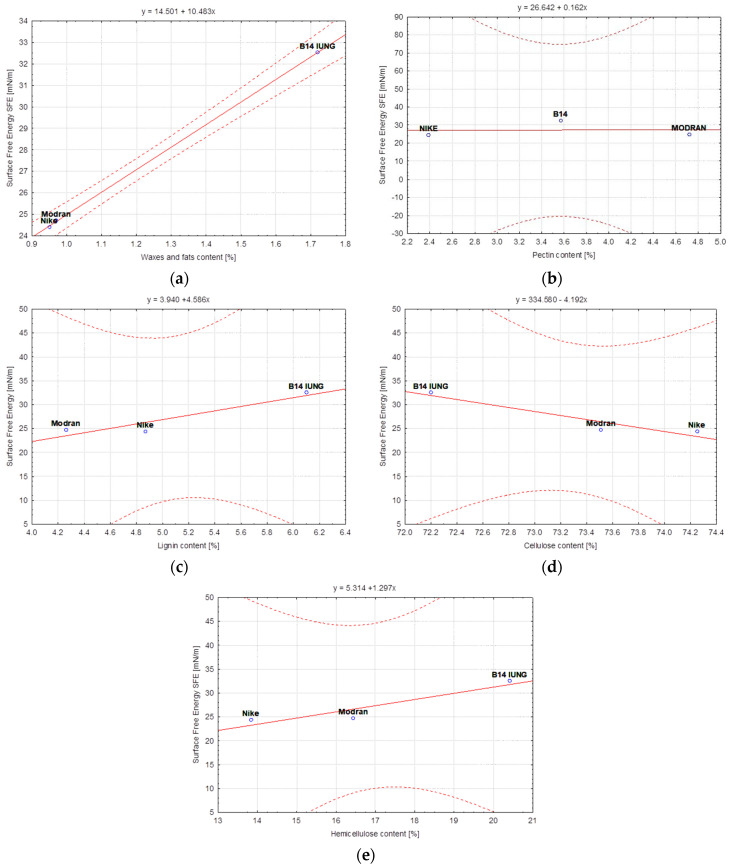
Variety of flax fibers—Dependence of Surface free energy [mN/m] vs.: (**a**) Waxes and fats content [%], (**b**) Pectin content [%], (**c**) Lignin content [%], (**d**) Cellulose content [%], (**e**) Hemicellulose content [%].

**Figure 9:**
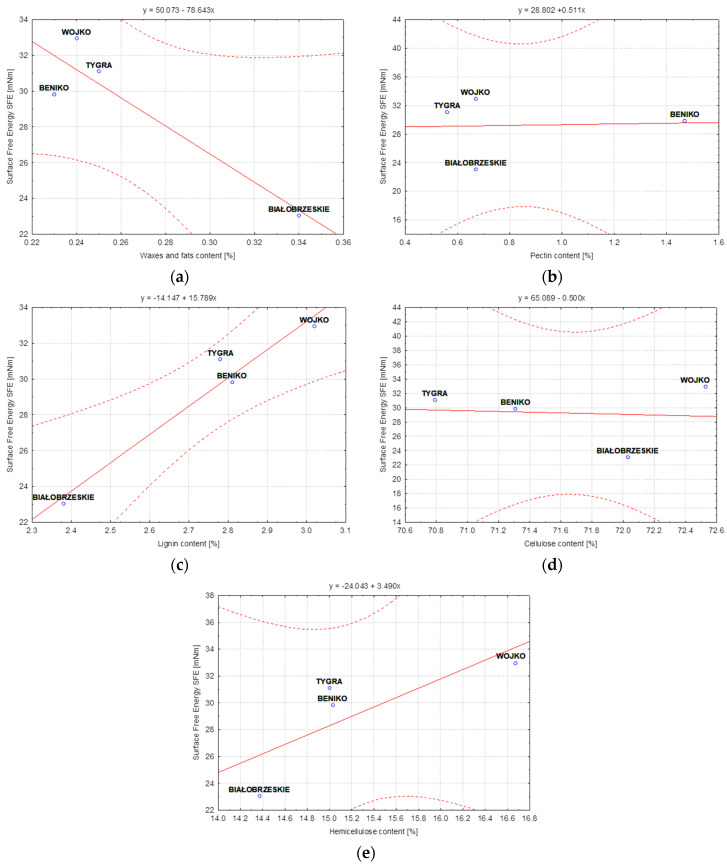
Variety of hemp fibers—Dependence of Surface free energy [mN/m] vs.: (**a**) Waxes and fats content [%], (**b**) Pectin content [%], (**c**) Lignin content [%], (**d**) Cellulose content [%], (**e**) Hemicellulose content [%].

**Figure 10:**
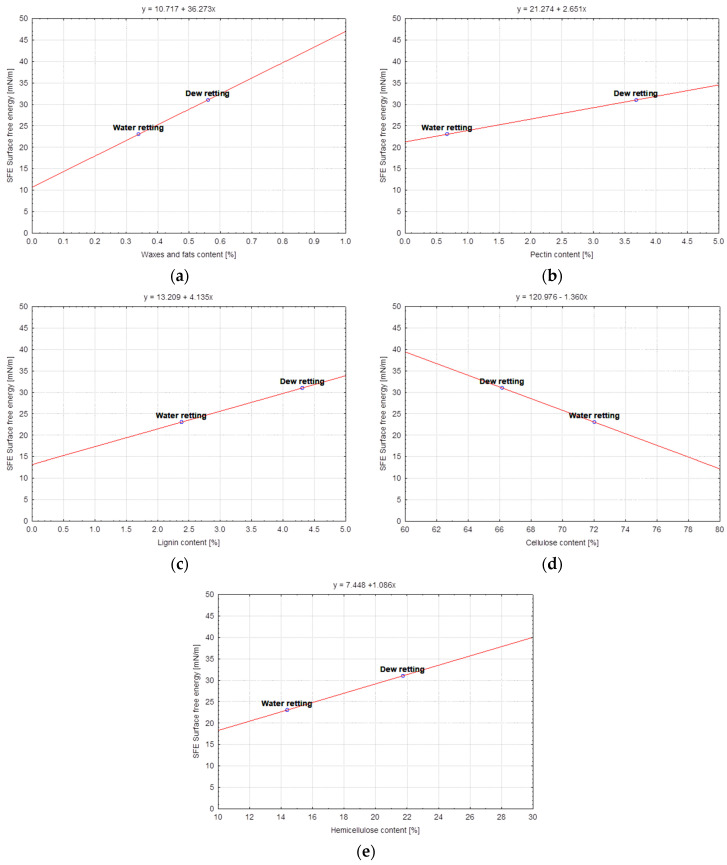
BIAŁOBRZESKIE variety hemp fibers—Dependence of Surface free energy [mN/m] vs.: (**a**) Waxes and fats content [%], (**b**) Pectin content [%], (**c**) Lignin content [%], (**d**) Cellulose content [%], (**e**) Hemicellulose content [%].

In this study, statistical analysis was performed using the STATISTICA software (8, TIBCO Software Inc., Palo Alto, CA, USA). The correlation approach was chosen for the model quality analysis due to the possibility of identifying a linear relationship between two variables. The strength of the correlation between the content of individual components of the discussed variants of flax and hemp fibers and the surface free energy was determined (Table 5) by calculation of Pearson’s linear correlation coefficients. The obtained results of the correlation coefficients were presented in the form of linear functional relationships with confidence intervals at the level of 95% (Figure 8, Figure 9 and Figure 10).

In the subsequent stages of the technological process applied for flax fiber, pectin sticking the monofilaments into bundles was removed, which is visible in the case of NIKE. In the case of MODRAN and B14, this regularity is not clear, resulting a weakening in the correlation between the content of this component and the surface free energy of the fiber (Table 5). Removal of pectin leads to easier access to other ingredients, such as waxes, fats, lignin and hemicellulose. The Pearson’s linear correlation coefficients determined the relationship between these components and the flax fiber free surface energy increased after each process. On the other hand, cellulose, which is the main component of the fiber, binds water molecules during the wet degumming processes resulting in a change in the fiber polarity. In consequence, this phenomenon caused a correlation between cellulose and surface free energy that reached a negative value. Nevertheless, after the last technological stage, i.e., the cottonization process, further removal of pectin took place resulting in a more effective separation of elementary fibers, which caused an increase in the specific fiber surface and its wettability. The correlation between cellulose and the surface free energy is still negative but the Pearson’s coefficient value is the highest in comparison to fibers tested after previous stages of technology.

In the case of a test of different varieties of water-retted hemp fibers, it should be noted that the process caused the washing out of pectin from the fiber effectively (Table 5), which resulted in easier access to other fiber chemical components such as, lignin, hemicellulose, waxes and fats, which have an effect on the strong correlation between the surface free energy and these components. However, the Pearson coefficient had negative value for waxes and fats. The washing out of the pectin from the hemp fibers during the water retting caused a lack of correlation between the surface free energy and the pectin. There was also no correlation between surface free energy and cellulose content in the hemp fibers extracted with the use of water retting from different plant varieties.

The slope of the regression line (Figure 10) for the hemp BIAŁOBRZESKIE variety takes positive values for the ratio of surface free energy to components such as waxes and fats, pectin, lignin and hemicellulose. This is due to the fact that the values of the content of the above-mentioned components are higher for the fiber obtained by the dew-retting method in the field than for the water-retting method (Table 3). The reverse is the case for the ratio of surface free energy to the cellulose content, where the slope of the regression line is negative (Figure 10d) because the cellulose content of the fiber extracted by water retting is higher than that of the cellulose extracted by the dew-retting method in the field (Table 3).

## 4. Conclusions

Bast fibers are materials with hydrophilic properties. This means that the fibers are characterized by their ability of sorption to water due to the high contents of cellulose and hemicellulose, which bind water molecules and determine the wettability of the fiber.

The research allowed us to conclude:

1. The surface free energy depends on the plant variety, which determines fibers chemical composition—The surface free energy of the decorticated flax fibers ranges from 22.72 mN/m (MODRAN) to 37.26 mN/m (NIKE) and depends on the plant variety.
2. The consecutive stages of the flax fiber refining process, i.e., hydrodynamic degumming of decorticated fibers and then cottonization, lead to the reorientation of the polar and dispersion character of the fiber and thus cause a change in the surface free energy of the fibers. In the case of NIKE on each consecutive stage, the differences between the polar and dispersion components of the fiber became smaller and led to a decrease in the value of surface free energy. In the case of the MODRAN fiber, the differences between the polar components were also reduced in the case of fibers obtained in the last stage of the technology, but to a lesser extent than in the case of NIKE, hence the surface free energy is approximately at the same level for the MODRAN fiber after each processing stage. The B14 fiber is not analogous to other flax fibers, the differences between the polar and dispersion components were more differentiated and the surface free energy was slightly reduced in the subsequent stages of the process. However, the results of the research on flax fibers allowed for the conclusion that the subsequent stages of the technological process do not have a statistically significant impact on the level of surface free energy of the processed fibers. Finally, the fibers after the final refinement stage, i.e., cottonization, are characterized by a surface free energy from 24.72 mN/m (MODRAN) to 32.53 mN/m (B14).
3. The values of fiber surface free energy tests showed the dependence of this parameter on the content of individual chemical components of the fiber, both in the case of flax and hemp. From the point of view of wettability, the most important aspects for bast fibers are the amount of cellulose and hemicellulose contained in the fiber. However, the hydrophobic ingredients such as lignin, waxes and fats in the fibers cannot be overlooked. The diversified proportion of hydrophilic and hydrophobic components in flax or hemp fibers is responsible for the differences in the surface free energy between fibers of different fibrous plant varieties. In the case of hemp fibers extracted by water retting, the WOJKO fiber had the highest surface free energy (32.95 mN/m). WOJKO fiber was characterized by a high content of hydrophobic components and the highest content of hydrophilic components as well, hence the difference between the polar and dispersion components was the highest of all the hemp fibers considered. The BIAŁOBRZESKIE fibers had the lowest content of hydrophobic components resulting in the lowest difference between the polar and non-polar components and thus the surface free energy of the BIAŁOBRZESKIE variety is the lowest (23.05 mN/m) among all the tested hemp fibers.
4. The surface free energy depends on the use of the defined method of retting—In the case of hemp fiber, the BIAŁOBRZESKIE variety influenced the wettability of the fiber. Water molecules bind to the hydrophilic groups of the fiber during water retting, causing changes in its polarity. Hemp fiber of the BIAŁOBRZESKIE variety extracted using water retting was therefore characterized by a lower surface free energy (23.05 mN/m) than the same fiber extracted with the use of dew retting of the straw (31.03 mN/m).
5. Statistical analysis confirmed that the surface free energy was strongly correlated with the content of individual components in the fibers. However, the intensity of the correlation between the fibers and the values of surface free energy, shown in the statistical analysis, proved that it can be weakened or strengthened as a result of subsequent processes in the technological chain and at the stage of straw retting.

Thus, the conclusion can be drawn that by selecting specific methods of retting the straw of fibrous plants or planning the design of treatment process, it is possible to shape the wettability of the fibers. Thus, the sorption properties of products made of these fibers depending on the needs determine whether it will be used for clothing products or intended for technical products.

Therefore, the following points are suggested for future work:

- Identification of the relationship of moisture management in textiles and values of surface free energy of the fibers,
- Selection of flax and hemp fibers in terms of their free surface energy value in order to improve moisture transport in the clothing,
- Selection of the appropriate variety and method of extraction of flax and hemp fibers for the production of biocomposites in terms of their surface free energy value and their ability to bond with the matrix,
- Carrying out chemical modification of flax and hemp fibers in order to increase the adhesion of fibers with the polymer matrix in biocomposites by increasing/decreasing the value of surface free energy.

## Figures and Tables

**Table 1:** Chemical composition of three varieties of flax fibers, obtained with the use of a technological chain consists of decortication, degumming and cottonization. Results are expressed as mean value and standard deviation (SD). Values sharing the same letter are not significantly different from each other (Tukey’s HSD, *p* < 0.05) [7].

Fibers	Waxes and Fats		Pectin		Lignin		Cellulose		Hemicellulose	
	[%]	SD	[%]	SD	[%]	SD	[%]	SD	[%]	SD
MODRAN										
Decortication	1.26	0.00	4.62 ^a^	0.16	4.00 ^a^	0.16	68.89	1.91	29.35	0.16
Wet degumming	0.69	0.07	4.41 ^a^	0.50	4.20 ^a^	0.16	75.54 ^a^	1.18	19.62	0.15
Cottonization	0.97	0.10	4.72 ^a^	0.39	4.26 ^a^	0.15	73.51 ^a^	0.98	16.44	0.23
NIKE										
Decortication	1.47	0.07	4.11	0.38	8.60	0.30	64.57	0.85	29.38	0.08
Wet degumming	0.76	0.00	3.56	0.27	4.46 ^a^	0.48	77.44	1.58	16.43	0.25
Cottonization	0.95	0.05	2.39	0.22	4.87 ^a^	0.51	74.25	0.20	13.84	0.06
B14										
Decortication	0.88	0.01	4.16	0.12	5.25	0.03	68.21	1.06	31.02	0.25
Wet degumming	1.33	0.01	5.43	0.28	6.69 ^a^	0.48	75.04	0.46	23.92	0.02
Cottonization	1.72	0.09	3.57	0.23	6.10 ^a^	0.01	72.20	0.47	20.41	0.09

Note: ^a^—represents the groups for which the mean values do not differ statistically at the assumed significance level α = 0.05.

**Table 2:** Chemical composition of hemp fibers coming from different plant varieties after water retting. Results are expressed as mean value and standard deviation (SD). Values sharing the same letter are not significantly different from each other (Tukey’s HSD, *p* < 0.05) [7].

Fibers	Waxes and Fats		Pectin		Lignin		Cellulose		Hemicellulose	
	[%]	SD	[%]	SD	[%]	SD	[%]	SD	[%]	SD
Water retting										
BENIKO	0.23 ^a^	0.01	1.47	0.09	2.81 ^a^	0.29	71.31 ^a^	1.32	15.03 ^a^	0.02
WOJKO	0.24 ^a^	0.04	0.67 ^a^	0.02	3.02 ^a^	0.31	72.53 ^a^	0.11	16.67	0.24
TYGRA	0.25 ^a^	0.04	0.56	0.00	2.78 ^a^	0.28	70.79 ^a^	0.13	15.00 ^a^	0.28
BIAŁOBRZESKIE	0.34	0.02	0.67 ^a^	0.02	2.38 ^a^	0.22	72.03 ^a^	0.22	14.37	0.29

Note: ^a^—represents the groups for which the mean values do not differ statistically at the assumed significance level α = 0.05.

**Table 3:** Chemical composition of hemp fibers, BIAŁOBRZESKIE variety, extracted from the straw with the use of water and dew retting. Results are expressed as mean value and standard deviation (SD). Values sharing the same letter are not significantly different from each other (Tukey’s HSD, *p* < 0.05) [7].

Fibers	Waxes and Fats		Pectin		Lignin		Cellulose		Hemicellulose	
	[%]	SD	[%]	SD	[%]	SD	[%]	SD	[%]	SD
BIAŁOBRZESKIE										
Dew retting	0.56 ^a^	0.14	3.68	0.19	4.31	0.04	66.16	0.48	21.72	0.12
Water retting	0.34 ^a^	0.02	0.67	0.02	2.38	0.22	72.03	0.22	14.37	0.29

Note: ^a^—represents the groups for which the mean values do not differ statistically at the assumed significance level α = 0.05.

**Table 4:** The characteristics of the main absorbance spectra in FTIR of flax and hemp fiber [7].

Bond	Vibration Type	Wavenumber [cm^−1^]	Remarks
O-H	Stretching	3100–3600	Cellulose, hemicellulose, lignin, pectin
C-H_3_	Stretching	2954–2970	Lignin
C-H, C-H_2_	Stretching	2915–2923; 2895–2897; 2841–2848	Cellulose, hemicellulose, lignin, pectin, waxes and fats
C=O	Stretching	1730–1736	Carboxylic acids, aldehydes, esters (pectin, lignin, waxes and fats)
O-H	Stretching	1615–1645	Adsorbed water
C=C Aromatic	Symmetrical Stretching	1593–1595; 1507–1508	Peaks characteristic of lignin
O-H and C-H_3_ and C-H_2_	Bending and Deforming	1461–1463 and 1461–1463; 1472–1473	Adsorbed water and Lignin and cellulose, hemicellulose, pectin, waxes and fats
COO	Stretching	1418–1420; 1424–1426	Acids (pectin)
C-H_3_	Symmetrical Deformation	1370–1373	Lignin
O-H	Bending	1332–1338	Cellulose, hemicellulose, lignin, pectin
CH_2_	Scissoring (bending)	1312–1314	Cellulose, hemicellulose
C-H	Bending	1271–1278	Peak characteristic for lignin
C-O	Stretching	1244–1246	Hemicellulose, pectin
C-H	Bending	1201–1204	Flax, hemp
C-O-C	Bending	1156–1161; 1051; 1020–1028	Cellulose, hemicellulose, pectin
C-O	Stretching	910–1125	Cellulose, hemicellulose, pectin
Β-Glycosidic bond	Stretching	893–897	Cellulose, hemicellulose, pectin

**Table 5:** Pearson’s linear correlation coefficients as the relation between the content of individual chemical components in the tested flax and hemp fibers and their surface free energy SFE.

	Waxes and Fats Content [%]	Pectin Content [%]	Lignin Content [%]	Cellulose Content [%]	Hemicellulose Content [%]
Flax fibers tested after each stage of processing					
SFE of flax fibers after decortication process [mN/m]	−0.04	−1.00	0.79	−0.71	0.40
SFE of flax fibers after wet degumming process [mN/m]	0.87	0.46	0.87	−0.10	0.49
SFE of flax fibers after cottonization process [mN/m]	1.00	0.04	0.93	−0.95	0.93
Hemp fibers extracted with the use of water retting					
SFE of hemp fibers after water-retting process [mN/m]	−0.92	0.05	0.98	−0.09	0.79

## Data Availability

Data are contained within the article.
